# Predictive value of advanced glycation end products for the development of post-infarction heart failure: a preliminary report

**DOI:** 10.1186/1475-2840-11-102

**Published:** 2012-08-21

**Authors:** Sergio Raposeiras-Roubín, Bruno K Rodiño-Janeiro, Beatriz Paradela-Dobarro, Lilian Grigorian-Shamagian, José M García-Acuña, Pablo Aguiar-Souto, Michel Jacquet-Hervet, María V Reino-Maceiras, Ezequiel Álvarez, José R González-Juanatey

**Affiliations:** 1 Servicio de Cardiología. Hospital Clínico Universitario de Santiago de Compostela, Santiago de Compostela, Spain; 2 Instituto de Investigación Sanitaria Santiago de Compostela, Santiago de Compostela, Spain; 3 Servicio de Cardiología. Hospital Meixoeiro, Vigo, Spain; 4 Departamento de Medicina, Universidad de Santiago de Compostela, Santiago de Compostela, Spain

**Keywords:** Advanced glycation end products, Heart failure, Myocardial infarct, Ventricular remodelling, Diabetes mellitus

## Abstract

**Background:**

Since post-infarction heart failure (HF) determines a great morbidity and mortality, and given the physiopathology implications of advanced glycation end products (AGE) in the genesis of myocardial dysfunction, it was intended to analyze the prognostic value of these molecules in order to predict post-infarction HF development.

**Methods:**

A prospective clinical study in patients after first acute coronary syndrome was conducted. The follow-up period was consisted in 1 year. In 194 patients consecutively admitted in the coronary unit for myocardial infarct fluorescent AGE levels were measured. The association between glycaemic parameters and the development of post-infarction HF were analyzed in those patients. Finally, we identified the variables with independent predictor value by performing a multivariate analysis of Hazard ratio for Cox regression.

**Results:**

Eleven out of 194 patients (5.6%) developed HF during follow-up (median: 1.0 years [0.8 - 1.5 years]). Even though basal glucose, fructosamine and glycated haemoglobin were significant predictive factors in the univariate analysis, after being adjusted by confounding variables and AGE they lost their statistical signification. Only AGE (Hazard Ratio 1.016, IC 95%: 1.006-1.026; *p*<0,001), together with NT-proBNP and the infarct extension were predictors for post-infarction HF development, where AGE levels over the median value 5-fold increased the risk of HF development during follow-up.

**Conclusions:**

AGE are an independent marker of post-infarction HF development risk.

## Background

Heart failure (HF) is a major cause of morbidity and mortality. Its incidence is increasing, in part as a secondary effect of the growing number of myocardial infarction survivor patients due to advances in drug therapy and cardiovascular interventions [1]. After myocardial infarction, physiological and anatomical ventricular changes occur. Left ventricular dilatation, eccentric hypertrophy, thinning of myocardial wall in the area of the scar and eventually left ventricular geometry alteration are aspects that define this process [2]. These changes are collectively known as ventricular remodelling, and they start a little after the myocardial infarction (even before any symptoms has been shown) as a progressive process that involves a worse prognosis for patients [3].

Ventricular remodelling is based on a neurohormonal model in which compensatory mechanisms of hormones and peptides acting in the kidney, as well as the peripheral vascular system and myocardium are implicated [4]. There is also an inflammatory reaction with release of cytokines, growth factors and reactive oxygen species production [5], which contributes to perpetuate ventricular dysfunction and could have important implications for prognosis.

Recent studies have shown the implication of hyperglycaemia in the development of HF [6], establishing an independent prognostic value for glycated haemoglobin (HbA1c) predicting the risk of HF in both diabetic and non-diabetic patients [7,8]. HbA1c is only an early glycation product [9]. However, there are no human studies demonstrating pathophysiological or prognostic implications for advanced glycation end products (AGE) in post-infarction HF.

AGE levels are increased in pro-inflammatory and oxidative stress states [10,11]. Either by their direct interaction with proteins, such as extracellular collagen, or by its interaction with its receptor (RAGE), AGE can lead to diastolic, systolic and vascular dysfunction [12]. Therefore, it could be hypothesized that, instead of acting as a single marker, these glycation end products might play an important role in post-infarction HF.

In this work, we analyze the prognostic value of both early (fructosamine and HbA1c) and advanced (AGE) glycation products in the development of post-infarction HF.

## Subjects and methods

### Study population

This is a prospective single centre study including all consecutive patients admitted with acute myocardial infarction (based on the universal definition of infarction [13]) in the coronary care unit from our hospital between October 2009 and January 2011, and who had survived to the acute coronary event during the hospital stay. Exclusion criteria included the presence of pregnancy, a history of HF, cardiomyopathy, moderate-severe valve disease, previous coronary artery disease, stroke, peripheral arterial disease, renal dysfunction on admission (defined by a rate of glomerular filtration rate by MDRD-4 <60 ml/min/1.73 m^2^), chronic liver disease, autoimmune or chronic inflammatory diseases, recent (last 3 weeks) infectious process, recent (last 3 weeks) treatment with corticosteroids or anti-inflammatory drugs, known tumour processes at the time of inclusion in the study, blood disorders and hospital admissions in the last month. As a result, 194 patients were included in the study after informed consent, according to rules approved by the Clinical Research Ethics Committee of Galicia (Spain).

### Protocol

The protocol study included a complete medical history, serum biochemistry and echocardiography within 24 hours. The diagnosis of diabetes mellitus (DM) was based on the latest diagnostic criteria established by the American Diabetes Association [14]. Left ventricular ejection fraction (LVEF) ≤ 45% was considered as depressed. Therapeutic strategy and pharmacological treatment were prescribed according to Clinical Practice Guidelines published by the European Society of Cardiology [15,16], always based on clinical judgment of cardiologists responsible for the patients in the coronary care unit.

### Laboratory parameters

AGE were measured by fluorescence spectrometry (Munch’s method [17]), taking into account the fluorescent property of some AGE, which emit strong fluorescence at 460 nm after being subjected to an excitation source at 360 nm. For this purpose, using samples of plasma of 80 μl in multiwell dark plates, AGE were measured by duplicate in a multi-mode reader (Synergy 2, Biotek) with a coefficient of variation of less than 8%. The results of these measurements were expressed in arbitrary fluorescence units (AU). Glycated haemoglobin (HbA1c) was determined by high performance liquid chromatography and fructosamine was measured by the enzymatic method GlyPro (with kits from Genzyme). NT-proBNP was measured by ELISA and cardiac troponin I was measured using an ultrasensitive kit.

### Monitoring and events

Patients were followed for a average period of 367.5 days (301.8 to 530.5). During follow-up, 4 patients (2.1%) died. Primary endpoint was defined as the readmission by HF, considering as such the need for hospitalization or stay in emergency room at least 24 hours and/or need for intravenous diuretic therapy. The diagnosis of HF were stabilized by a cardiologist blind to the study, based on clinical criteria and a structural and/or functional heart anomaly detectable by echocardiography, according to the diagnostic criteria for HF proposed by the European Society of Cardiology [18].

### Statistical analysis

In order to accomplish the analysis of data, we used SPSS (SPSS Inc, Chicago, Illinois, version 17.0). Categorical variables were expressed as frequencies and percentages. With regard to continuous variables, the assumption of normality was tested with the Kolmogorov-Smirnov test. The variables that follow a normal distribution were expressed as mean ± standard deviation. The remaining variables were expressed as median and interquartile range. The association between categorical variables were tested using the Chi-square test and the relationship between quantitative variables was determined by Pearson correlation. The comparison of dichotomous categorical quantitative variables was carried out with the Student’s *t*-test (when normality condition was reached) or the non-parametric "U" test by Mann–Whitney (if the no-normal condition was the one satisfied). To study the independence of the association between fluorescent AGE and post-infarction HF development, those variables with clinical or statistical significance for univariate analysis were included in a Cox regression model (backward stepwise Hazard analysis). Since the number of events was low (*n* = 11), variables that could lead to interactions (eg, fructosamine with glycated hemoglobin) were avoided in order to improve accuracy getting results. Based on this, we took into account the following variables for Hazard Ratio analysis: age (years), diabetes mellitus, heart rate (bpm), depressed left ventricular ejection fraction (LVEF≤45%), haemoglobin on admission (g/dL), troponin I peak (ng/dL), NTproBNP (for 100 pg/mL), HbA1c (%) and fluorescent AGE (AU). We considered significant *p* values < 0.05.

## Results

### Baseline characteristics and clinical considerations

In Table 1, demographic, clinical and analytical characteristics of patients, as well as their therapeutic manipulations, have been summarized. Based on HF development during follow-up, patients were classified in two groups. As can be seen, at the time of hospital admission, patients who developed post-infarction HF presented worst killip class, increased heart rate, greater myocardial damage (expressed as higher troponin I peak) and higher systolic ventricular dysfunction, lower haemoglobin levels and increased serum concentration of NT-proBNP and glycaemic control parameters (although there were no differences depending on the presence or absence of DM). There were no significant differences neither in percutaneous intervention nor in coronary artery bypass grafting. The pharmacological therapy was very similar in both groups. Only one difference was observed; the anti-aldosterone drugs were more used in patients with post-infarction HF, secondary to the existence of higher systolic ventricular dysfunction.

**Table 1:** Baseline characteristics of the study population, stratified by groups according to whether or not they developed HF during the follow-up period

	**Total**	**Heart failure**		
		**YES**	**NO**	***p***
***Demographic data***				
Age (years)	62.8 ± 13.3	67.4 ± 11.6	62.6 ± 13.4	0.244
Female, %	24.7	27.3	35.0	0.841
***Medical history***				
BMI (Kg/m^2^)	27.0±3.3	25.7±3.9	27.1±3.5	0.225
Current smoking, %	34.5	27.3	35.0	0.703
Diabetes, %	26.8	36.4	26.2	0.461
Hypertension, %	47.9	63.6	47.0	0.283
Dyslipidemia, %	44.8	45.5	44.7	0.957
***On admission data***				
STEMI, %	52.1	72.7	50.8	0.158
Killip class ≥ II, %	13.9	36.4	12.6	**0.027**
Systolic blood pressure (mmHg)	137.0 ± 26.4	135.8 ± 25.7	137.1 ± 26.5	0.881
Heart rate (bpm)	75.2 ± 18.8	93.8 ± 16.4	74.1 ± 18.4	**0.001**
Atrial fibrillation, %	8.2	18.2	7.7	0.217
Haemoglobin (g/dL)	14.3 ± 1.6	13.1 ± 1.5	14.4 ± 1.6	**0.011**
Leukocytes (10^3^/μL)	9.8 ± 4.0	9.6 ± 4.1	10.9 ± 4.0	0.314
Creatinine (mg/dL)	0.95 ± 0.15	0.99 ± 0.12	0.95 ± 0.16	0.396
***Laboratory parameters***				
TPI peak (ng/dL)	17.1 (5.6-68.2)	114.7 (23.5-171.5)	15.3 (5.2-59.4)	**0.024**
NT-proBNP (pg/mL)	801.5 (352.7-1779.3)	2938.0 (1400.0-3655.0)	769.5 (320.5-1663.2)	**0.011**
***Glycation products***				
Glucose (mg/dL)	152.9 ± 74.1	203.0± 96.7	149.9 ± 71.7	**0.021**
Fructosamine (mg/dL)	192.4 ± 71.5	243.5 ± 95.3	188.5 ± 68.2	**0.019**
HbA1c (%)	6.2 ± 1.4	7.1 ± 2.3	6.1 ± 1.3	**0.017**
AGE fluorescent (AU)	57.3 ± 45.0	95.9 ± 83.1	54.9 ± 40.9	**0.003**
***Procedural characteristics***				
LVEF ≤ 45%, %	19.6	54.5	17.5	**0.003**
Multivessel disease, %	52.1	63.6	51.4	0.429
PCI, %	82.0	72.7	82.5	0.412
CABG, %	5.7	9.1	5.5	0.617
Complete revascularization, %	80.2	37.5	82.3	**0.002**
***Medical treatment***				
Acetylsalicylic acid	100.0	100.0	100.0	-
Clopidogrel	99.0	98.9	100.0	0.722
Anti-IIb-IIIA	19.1	9.1	19.7	0.386
B-blockers	86.1	90.8	85.8	0.634
ACE Inhibitors-ARAII	89.7	89.6	90.9	0.767
Anti-aldosterone medication	20.1	63.6	17.5	**<0.001**
Statins	94.9	90.0	95.2	0.463
Insulin	5.7	18.2	4.9	0.065
Oral anti-diabetics	11.8	20.0	11.3	0.408

*AGE*: advanced glycation end products; *ARAII*: angiotensin II receptor antagonists; *BMI*: body mass index; *CABG*: coronary artery bypass graft; *HbA1c*: glycated haemoglobin; *LVEF*: left ventricular ejection fraction; *STEMI:* ST segment elevation myocardial infarction; *PCI*: percutaneous coronary intervention; *ACE*: angiotensin-converting enzyme; *NT-proBNP*: N-terminal fragment of brain natriuretic peptide; *TPI*: troponin I peak.

### Clinical significance of glycation parameters

Glucose, fructosamine and HbA1C presented a strong interrelationship. However, fluorescent AGE did only show correlation with fructosamine (r = 0.169; *p* = 0.045). There was no relationship neither between fluorescent AGE and HbA1c (r = 0.144; *p* = 0.061) nor between AGE and glucose (r = 0.108; *p* = 0.136). Considering the association with DM (Table 2), all parameters were significantly increased in diabetic patients; only fluorescent AGE presented the same value in diabetic and non-diabetic patients.

**Table 2:** Glycaemic parameters association with diabetes

	**Diabetes**	**N**	**Media**	**Standard deviation**	***p***
**Glucose (mg/dL)**	YES	52	204.6	95.9	**<0.001**
	NO	142	134.0	53.2	
**Fructosamine (mg/dL)**	YES	40	254.9	85.4	**<0.001**
	NO	101	167.6	46.1	
**HbA1c (%)**	YES	48	7.2	1.8	**<0.001**
	NO	121	5.6	0.8	
**AGE (AU)**	YES	52	67.3	60.8	0.132
	NO	142	53.5	37.1	

*AGE*: advanced glycation end products; *HbA1c*: glycated haemoglobin.

In Table 3, patients have been grouped based on high and low levels of early (HbA1c) and late (fluorescent AGE) glycation products. As can be seen, patients with increased levels of HbA1c presented a higher cardiovascular risk profile (older, higher BMI and percentage of DM, hypertension and dyslipidemia). This resulted in a greater percentage of multivessel disease, with a trend toward a higher atrial fibrillation percentage and higher Killip class on admission. As opposed to this, increased levels of fluorescent AGE were only associated with a higher percentage of hypertension and a lower rate of myocardial infarction with ST segment elevation. No correlation with DM, multivessel disease or ventricular dysfunction was observed, considering median as the cut-off point (for HbA1c and fluorescent AGE).

**Table 3:** Patients’ characteristics according to high or low levels of early (HbA1c) or end (fluorescent AGE) glycation products, considering median as cut off point

	**AGE**			**HbA1c**		
	**HIGH**	**LOW**	***p***	**HIGH**	**LOW**	***p***
***Demographic data***						
Age (years)	62.7 ± 14.7	62.9 ± 11.7	0.913	67.5 ± 12.5	58.1 ± 12.7	**0.001**
Female, %	22.9	26.5	0.560	28.9	21.5	0.272
***Medical history***						
BMI (Kg/m^2^)	27.1 ± 3.6	26.9 ± 3.6	0.839	27.6 ± 4.0	26.2 ± 3.0	**0.020**
Current smoking, %	39.6	29.6	0.290	24.4	39.2	0.116
Diabetes, %	28.1	25.5	0.681	43.3	11.4	**0.001**
Hypertensión, %	55.2	40.8	**0.045**	60.0	35.4	**0.001**
Dyslipemia, %	51.0	38.0	0.086	51.1	32.9	**0.017**
***On admission data***						
STEMI, %	45.8	58.2	0.086	48.9	53.2	0.579
Killip class ≥ II, %	11.5	16.3	0.327	18.9	8.9	0.062
Systolic blood pressure (mmHg)	137.9 ± 25.1	136.0 ± 27.8	0.603	141.2 ± 29.2	133.3 ± 22.2	0.050
Heart rate (bpm)	74.7 ± 18.3	75.7 ± 19.4	0.714	78.1 ± 19.8	72.2 ± 16.0	**0.035**
Atrial fibrillation, %	7.3	9.2	0.632	13.3	5.1	0.067
Haemoglobin (g/dL)	14.3 ± 1.8	14.4 ± 1.4	0.829	14.1 ± 1.7	14.5 ± 1.4	0.104
Leukocytes (10^3^/μL)	10.6 ± 3.8	10.9 ± 4.1	0.622	10.5 ± 3.8	10.9 ± 4.1	0.453
Creatinine (mg/dL)	0.97 ± 0.15	0.93 ± 0.17	0.126	0.96 ± 0.15	0.94 ± 0.16	0.418
***Laboratory parameters***						
TPI peak (ng/dL)	13.9 (6.4-70.1)	22.2 (4.5-69.4)	0.700	17.3 (6.2-85.6)	17.0 (5.1-63.2)	0.649
NT-proBNP (pg/mL)	783.0 (342.0-1712.7)	826.0 (376.5-1780.7)	0.348	1173.0 (450.2-2376.5)	568.5 (225.8-1235.2)	0.128
***On admission data***						
STEMI, %	15.6	23.5	0.169	23.3	15.2	0.183
Killip class ≥ II, %	58.3	45.9	**0.084**	61.1	39.2	**0.005**

Abbreviations as in Table 1.

### Glycation parameters and their association with post-infarction HF

**Figure 1:**
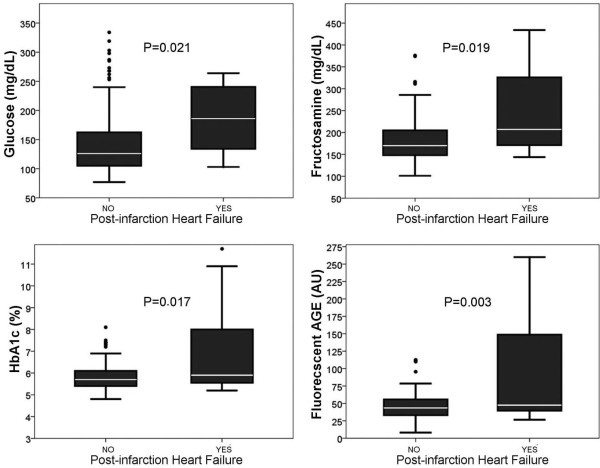
Levels of glucose, fructosamine, HbA1c and fluorescent AGE in patients who have suffered post-infarction HF during a median follow-up of 1 year compared with patients who have not developed HF.

**Figure 2:**
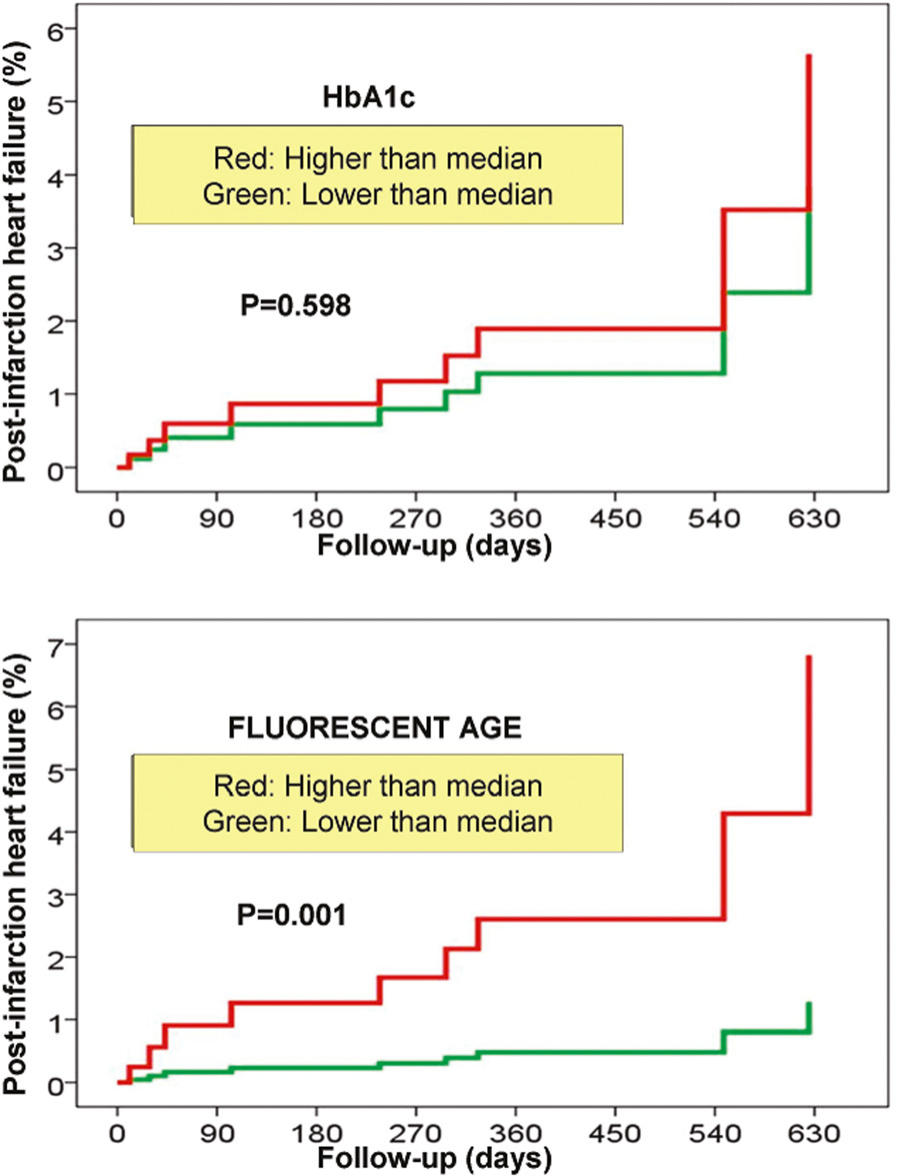
Cumulative incidence curves for HF after acute myocardial infarction for high and low levels of glycated haemoglobin or fluorescent AGE, adjusted for age, diabetes mellitus, systolic function, heart rate, haemoglobin levels, troponin peak and NT-proBNP levels.

Figure 1 shows the association of glucose, fructosamine, HbA1c and AGE plasmatic basal levels with HF development during follow-up. Eleven patients developed HF during follow-up (Table 4). A multivariate analysis was performed in order to determine the behaviour of each independent predictor of glycaemic control parameters (Table 5). As a means to get this, variables that were significantly associated with post-infarction HF development were taken into account (Table 1). Moreover, those variables with clinical significance documented on previous studies (GISSI [1] and PEACE [19]) were also included, even though they did not present statistical significance. After performing the multivariate analysis and adjusting for confounding variables, results, as reflected in Table 5, showed that troponin I peak, NT-proBNP levels and fluorescent AGE were the only variables that remained as independent predictors of post-infarction HF development. HbA1c lost its predictive value after being adjusted by the presence of DM and fluorescent AGE, among other variables. In and identical manner, two alternative models of the same multivariate analysis were developed. On one of these analyses, HbA1c was replaced by fructosamine and on the other one by glucose levels at admission. Parameters were separated in the analysis to avoid interaction between those variables and HbA1c, given the strong positive correlation showed. Results were similar to the ones obtained for HbA1c, remaining only fluorescent AGE, together with troponin I peak and NT-proBNP, as independent predictors of HF development. The HR ratio for fructosamine was 1.007 (95% CI: 0.989 to 1.025, *p* = 0.440) and for basal glucose 1.003 (95% CI: 0.991 to 1.014, *p* = 0.666). Figure 2 shows the fitted curves for HF development during follow-up where AGE and HbA1c values were above the median. As can be observed, high levels of AGE, but not high levels of HbA1c, can be used to predict HF post-infarction development (HR 5.467, 95% CI: 1.015 to 29.443, *p* = 0.048).

**Table 4:** Eleven patients developed post-infarction HF in the follow-up time indicated

**Post-infarction HF (cases)**	**Time of onset (days)**
1	11
2	29
3	35
4	43
5	103
6	233
7	237
8	326
9	468
10	548
11	625

**Table 5:** Results of the adjusted multivariate analysis for prediction of HF development during the follow-up period after myocardial infarction

**Variables**	**Hazard ratio**	**CI 95%**	***p***
**Age (years)**	1.046	0.964 – 1.135	0.284
**Diabetes mellitus**	0.526	0.053 – 5.267	0.585
**Heart rate (bpm)**	1.012	0.971 – 1.054	0.572
**LVEF ≤ 45%**	1.643	0.251 – 10.758	0.605
**Hb on admission (g/dL)**	0.957	0.564 – 1.624	0.871
**TPI peak (ng/dL)**	1.006	1.001 – 1.011	**0.025**
**NTproBNP (for 100 pg/mL)**	1.030	1.006 – 1.054	**0.015**
**HbA1c (%)**	1.153	0.679 – 1.955	0.598
**Fluorescent AGE (AU)**	1.016	1.007 – 1.026	**0.001**

*AGE*: advanced glycation end products; *Hb*: haemoglobin; *HbA1c*: glycated haemoglobin; *LVEF*: left ventricular ejection fraction; *NT-proBNP*: N-terminal fragment of brain natriuretic peptide; *TPI:* troponin I.

## Discussion

The most important finding of our study was that fluorescent AGE [detectable in plasma by 360/460 nm (exc./em.) fluorescence] is an independent and predictive biomarker for HF development risk after an acute myocardial infarct, whereas glycation precursors such as glycated haemoglobin lost their predictive value after a multivariate statistical adjustment. High AGE levels (over the median value) 5-fold increased the risk of post-infarction HF during the follow-up period, regardless of age, DM presence and glycaemic control, infarct’s seriousness (ventricle dysfunction and troponin elevation) and other biomarkers such as NT-proBNP. Although HbA1c, fructosamine and basal glucose, were significantly associated with a higher post-infarction HF rate in the univariate analysis, they lost their significance after being adjusted with other confounding variables, like AGE, in the multivariate analysis.

Within the last few years it has been reported the relation between hyperglycaemia and HbA1c and a higher risk of HF, both in diabetic and non-diabetic patients [6-8]. This suggests that maintained hyperglycaemia plays an important role as a myocardial deleterious agent [11] and that advanced glycation is one of the main action mechanisms [10]. The role played by the AGE-RAGE axis in the onset of HF has been studied [12,20,21], laying the foundations for the link between hyperglycaemia and HF.

AGE levels increase in the context of maintained hyperglycaemia [9] and they trigger intracellular signalling pathways that activate NF-κB transcription factor, promoting oxidative stress [10], which also contributes to AGE formation [11]. AGE lead to both diastolic and systolic dysfunction [12]. There are three reported molecular mechanisms for diastolic dysfunction mediated by AGE: 1) AGE establish cross links between matrix proteins decreasing their flexibility and promoting myocardial stiffness [22]. 2) Through the activation of their receptors (AGE-RAGE axis), AGE induce fibrosis by up-regulation of transforming growth factor beta [23]. 3) AGE-RAGE axis activation also may influence intracellular calcium homeostasis in cardiomyocytes, increasing repolarisation period [24]. Regarding systolic dysfunction, it stands out, by its clinical repercussion, the acceleration of the coronary artery disease progression. AGE-RAGE interaction may induce atherosclerosis, thrombosis and vasoconstriction [12]. AGE promote endothelial dysfunction secondary to a reduced nitric oxide bioavailability mediated by free radicals [25] and they have shown to increase endothelial permeability *in vitro*[26]. Endothelial dysfunction is involved in the early stages of atherosclerosis and may trigger endothelium repair by endothelial progenitor cells. However, AGE have demonstrated to promote apoptosis and impair functions of endothelial progenitor cells in culture [27] and also to reduce growth and migration of mesenchymal stem cells [28]. AGE can establish cross links with cholesterol-LDL particles resulting in the formation of more atherogenic molecules by increasing their affinity to macrophage receptors and enhancing foam cells formation [29]. AGE may also induce systolic dysfunction by reducing intracellular calcium levels [24] which conducts to a reduction of myocardial contractility. Taken all these considerations into account, the AGE-RAGE axis accelerates post-infarct myocardium remodelling, generating a deleterious feedback mechanism. In fact, the relation between the rennin-angiotensin-aldosterone system, a relevant system in ventricular remodelling and with demonstrated pathophysiological implications, with AGE-RAGE axis has been suggested [30]. So, angiotensin converting enzyme inhibitors and angiotensin II receptor antagonists reduced plasma AGE levels [31]. In contrast, it has been demonstrated that AGE promote angiotensin II formation [32]. Other possible ways to regulate AGE-RAGE axis activity have been explored. The most physiological one would be to increase physical activity in an attempt to reduce AGE-RAGE axis activity. A preliminary study has shown a reduction of soluble RAGE levels and an inverse correlation between these levels and paraoxonase-1 activity (an antioxidative enzyme) after an increasing physical activity intervention [33]. However, alagebrium, an AGE-breaker, has not shown to improve physical exercise tolerance \and other secondary endpoints in patients with heart failure in the BENEFICIAL clinical trial [34]. Even though, it is clear that AGE can play a role in post-infarction HF, being crucial elements of ventricular remodelling. However, to our knowledge, this is the first study to analyze the relationship between AGE levels and post-infarction HF.

Therefore, it is reasonable to think that all the data relating glycaemic and HbA1c with HF development risk [5-8] could be explained based on the AGE-RAGE axis, since the glucose-fructosamine-HbA1c pathway ends in AGE [11] as pentosidine or carboxymethyl-lysine and these molecules can mediate myocardial remodelling towards cardiac dysfunction. In fact, in this study, glucose, fructosamine and HbA1c were predictors of post-infarction HF in the univariate analysis, but after the adjustment by confounding variables and AGE all of them lost their significance. This result suggests that their predictive value is influenced by some of the other variables (probably AGE).

### Clinical implications

The higher level of AGE found in patients with post-infarction HF showed the pathophysiological role that these molecules can play in ventricular remodelling. This means that, rather than a simple risk biomarker after an acute myocardial infarction, AGE can be a new etiological way to focus therapeutic research to reduce the harmful effects of remodelling. In fact, there are some blockers of the AGE-RAGE axis that have been studied in animals. The best known are aminoguanidine, an AGE formation inhibitor [35], and alagebrium (ALT-711), an AGE breaker [36]. Both molecules have been tested in animals showing an improvement of myocardial compliance [35] and an enhancement of cardiac function in animals with contractile dysfunction [36]. The effect of alagebrium on diastolic dysfunction has also been studied in humans [36,37]. In the DIAMOND trial 23 stable patients with diastolic dysfunction were treated with alagebrium. After 16 weeks, left ventricle mass was reduced and diastolic function was improved [37]. The PEDESTAL trial studied the effects of alagebrium in HF patients with depressed systolic function (LVEF < 45%) and the preliminary results showed a tendency to improve systolic function [38]. Our work opens the field to study the possible role of AGE-RAGE axis blockers in post-infarction HF development prevention.

### Limitations

Despite the impact and enthusiasm that our results can generate, we are aware of the limitations of our study and they should be taken into account for the interpretation of the results. Mainly, we must consider that our study population, which included all patients admitted for acute coronary syndrome in the coronary care unit within 15 months, was subjected to strict inclusion/exclusion criteria to eliminate possible interfering variables, thus limiting the extrapolation of our results to the real world of acute myocardial infarction patients. On the other hand, this meant a significant reduction in our sample size (n=194) and, considering it along with the inclusion of very selective population with a lower cardiovascular risk, it determines the statistical power of the analysis. Furthermore, some analytical parameters were not determined in some of the patients. Hence, AGE were measured in 100% population, whereas HbA1c was measured in 87.2%, fructosamine in 72.7% and NT-proBNP in 79.4%. On the other hand, in our study AGE were measured by Much’s method, which means that an unspecified mixture of different fluorescent AGE were detected in the measurement, but non-fluorescent AGE were not considered. Despite all this, we believe that our results maintain the pathophysiological and clinical implications exposed and open the field to future investigations.

## Conclusions

AGE are independent biomarker for risk of post-infarction HF. It can be presumed that the pathophysiological base lays in the link between maintained hyperglycaemia and ventricular remodelling. Future investigations should confirm these findings and elucidate the pathophysiological and therapeutic implications.

## Abbreviations

AGE: Advanced Glycation End product; DM: Diabetes Mellitus; HbA1c: Glycated Haemoglobin; HF: Heart Failure; LVEF: Left Ventricular Ejection Fraction; NT-proBNP: N-Terminal pro-Brain Natriuretic Peptide; MDRD-4: Modification of Diet in Renal Disease 4; RAGE: Receptor for Advanced Glycation End-products.

## Competing interests

There is no competing interests that could be perceived as prejudicing the impartiality of the research reported.

## Authors’ contributions

Design: SPR, BKRJ, LGS, JMGA, EÁ, JRGJ. Conduct/data collection: SRR, BKRJ, BPD, LGS, JMGA, PAS, MJH, MVRM, EÁ. Analysis: SRR, BKRJ, BPD, LGS, EÁ. Writing manuscript: SRR, BKRJ, LGS, EÁ, JRGJ. All authors read and approved the final manuscript.

## References

[B1] Macchia A Levantesi G Marfisi RM Franzosi MG Maggioni AP Nicolosi GL Schweiger C Tavazzi L Tognoni G Valagussa F Determinants of late-onset heart failure in myocardial infarction survivors: GISSI Prevenzione trial results Rev Esp Cardiol 2005 58 11 1266 1272 10.1157/13080953 16324579

[B2] Gajarsa JJ Kloner RA Left ventricular remodeling in the post-infarction heart: a review of cellular, molecular mechanisms, and therapeutic modalities Heart Fail Rev 2011 16 1 13 21 10.1007/s10741-010-9181-7 20623185

[B3] Cohn JN Ferrari R Sharpe N Cardiac remodeling–concepts and clinical implications: a consensus paper from an international forum on cardiac remodeling. Behalf of an International Forum on Cardiac Remodeling J Am Coll Cardiol 2000 35 3 569 582 10.1016/S0735-1097(99)00630-0 10716457

[B4] Roig Minguell E Clinical use of markers of neurohormonal activation in heart failure Rev Esp Cardiol 2004 57 4 347 356 10.1157/13059727 15104989

[B5] Dominguez Rodriguez A Abreu Gonzalez P Garcia Gonzalez MJ Ferrer Hita J Association between serum interleukin 10 level and development of heart failure in acute myocardial infarction patients treated by primary angioplasty Rev Esp Cardiol 2005 58 6 626 630 10.1157/13076414 15970117

[B6] Held C Gerstein HC Yusuf S Zhao F Hilbrich L Anderson C Sleight P Teo K Glucose levels predict hospitalization for congestive heart failure in patients at high cardiovascular risk Circulation 2007 115 11 1371 1375 10.1161/CIRCULATIONAHA.106.661405 17339550

[B7] Pazin-Filho A Kottgen A Bertoni AG Russell SD Selvin E Rosamond WD Coresh J HbA 1c as a risk factor for heart failure in persons with diabetes: the Atherosclerosis Risk in Communities (ARIC) study Diabetologia 2008 51 12 2197 2204 10.1007/s00125-008-1164-z 18828004 PMC2848756

[B8] Matsushita K Blecker S Pazin-Filho A Bertoni A Chang PP Coresh J Selvin E The association of hemoglobin a1c with incident heart failure among people without diabetes: the atherosclerosis risk in communities study Diabetes 2010 59 8 2020 2026 10.2337/db10-0165 20484138 PMC2911067

[B9] Wolffenbuttel BH Giordano D Founds HW Bucala R Long-term assessment of glucose control by haemoglobin-AGE measurement Lancet 1996 347 9000 513 515 10.1016/S0140-6736(96)91141-1 8596270

[B10] Basta G Lazzerini G Massaro M Simoncini T Tanganelli P Fu C Kislinger T Stern DM Schmidt AM De Caterina R Advanced glycation end products activate endothelium through signal-transduction receptor RAGE: a mechanism for amplification of inflammatory responses Circulation 2002 105 7 816 822 10.1161/hc0702.104183 11854121

[B11] Giacco F Brownlee M Oxidative stress and diabetic complications Circ Res 2010 107 9 1058 1070 10.1161/CIRCRESAHA.110.223545 21030723 PMC2996922

[B12] Hartog JW Voors AA Bakker SJ Smit AJ van Veldhuisen DJ Advanced glycation end-products (AGEs) and heart failure: pathophysiology and clinical implications Eur J Heart Fail 2007 9 12 1146 1155 10.1016/j.ejheart.2007.09.009 18023248

[B13] Thygesen K Alpert JS White HD Jaffe AS Apple FS Galvani M Katus HA Newby LK Ravkilde J Chaitman B Universal definition of myocardial infarction Circulation 2007 116 22 2634 2653 10.1161/CIRCULATIONAHA.107.187397 17951284

[B14] American Diabetes Association Diagnosis and classification of diabetes mellitus Diabetes Care 2010 34 Suppl 1 S62 S69 10.2337/dc11-S062 PMC3006051 21193628

[B15] Bassand JP Hamm CW Ardissino D Boersma E Budaj A Fernandez-Aviles F Fox KA Hasdai D Ohman EM Wallentin L Guidelines for the diagnosis and treatment of non-ST-segment elevation acute coronary syndromes Eur Heart J 2007 28 13 1598 1660 17569677 10.1093/eurheartj/ehm161

[B16] Van de Werf F Bax J Betriu A Blomstrom-Lundqvist C Crea F Falk V Filippatos G Fox K Huber K Kastrati A Management of acute myocardial infarction in patients presenting with persistent ST-segment elevation: the task force on the management of ST-segment elevation acute myocardial infarction of the European society of cardiology Eur Heart J 2008 29 23 2909 2945 19004841 10.1093/eurheartj/ehn416

[B17] Munch G Keis R Wessels A Riederer P Bahner U Heidland A Niwa T Lemke HD Schinzel R Determination of advanced glycation end products in serum by fluorescence spectroscopy and competitive ELISA Eur J Clin Chem Clin Biochem 1997 35 9 669 677 9352229 10.1515/cclm.1997.35.9.669

[B18] Dickstein K Cohen-Solal A Filippatos G McMurray JJ Ponikowski P Poole-Wilson PA Stromberg A van Veldhuisen DJ Atar D Hoes AW ESC guidelines for the diagnosis and treatment of acute and chronic heart failure 2008: the Task Force for the diagnosis and treatment of acute and chronic heart failure 2008 of the European Society of Cardiology. Developed in collaboration with the Heart Failure Association of the ESC (HFA) and endorsed by the European Society of Intensive Care Medicine (ESICM) Eur J Heart Fail 2008 10 10 933 989 10.1016/j.ejheart.2008.08.005 18826876

[B19] Lewis EF Solomon SD Jablonski KA Rice MM Clemenza F Hsia J Maggioni AP Zabalgoitia M Huynh T Cuddy TE Predictors of heart failure in patients with stable coronary artery disease: a PEACE study Circ Heart Fail 2009 2 3 209 216 10.1161/CIRCHEARTFAILURE.108.820696 19808342 PMC3009573

[B20] Koyama Y Takeishi Y Arimoto T Niizeki T Shishido T Takahashi H Nozaki N Hirono O Tsunoda Y Nitobe J High serum level of pentosidine, an advanced glycation end product (AGE), is a risk factor of patients with heart failure J Card Fail 2007 13 3 199 206 10.1016/j.cardfail.2006.11.009 17448417

[B21] Hartog JW Voors AA Schalkwijk CG Scheijen J Smilde TD Damman K Bakker SJ Smit AJ van Veldhuisen DJ Clinical and prognostic value of advanced glycation end-products in chronic heart failure Eur Heart J 2007 28 23 2879 2885 10.1093/eurheartj/ehm486 17986469

[B22] Aronson D Cross-linking of glycated collagen in the pathogenesis of arterial and myocardial stiffening of aging and diabetes J Hypertens 2003 21 1 3 12 10.1097/00004872-200301000-00002 12544424

[B23] Cipollone F Iezzi A Fazia M Zucchelli M Pini B Cuccurullo C De Cesare D De Blasis G Muraro R Bei R The receptor RAGE as a progression factor amplifying arachidonate-dependent inflammatory and proteolytic response in human atherosclerotic plaques: role of glycemic control Circulation 2003 108 9 1070 1077 10.1161/01.CIR.0000086014.80477.0D 12912808

[B24] Petrova R Yamamoto Y Muraki K Yonekura H Sakurai S Watanabe T Li H Takeuchi M Makita Z Kato I Advanced glycation endproduct-induced calcium handling impairment in mouse cardiac myocytes J Mol Cell Cardiol 2002 34 10 1425 1431 10.1006/jmcc.2002.2084 12393002

[B25] Soro-Paavonen A Zhang WZ Venardos K Coughlan MT Harris E Tong DC Brasacchio D Paavonen K Chin-Dusting J Cooper ME Advanced glycation end-products induce vascular dysfunction via resistance to nitric oxide and suppression of endothelial nitric oxide synthase J Hypertens 2010 28 4 780 788 10.1097/HJH.0b013e328335043e 20186099

[B26] Wang J Liu H Chen B Li Q Huang X Wang L Guo X Huang Q RhoA/ROCK-dependent moesin phosphorylation regulates AGE-induced endothelial cellular response Cardiovasc Diabetol 2012 11 7 10.1186/1475-2840-11-7 22251897 PMC3280169

[B27] Li H Zhang X Guan X Cui X Wang Y Chu H Cheng M Advanced glycation end products impair the migration, adhesion and secretion potentials of late endothelial progenitor cells Cardiovasc Diabetol 2012 11 1 46 10.1186/1475-2840-11-46 22545734 PMC3403843

[B28] Yang K Wang XQ He YS Lu L Chen QJ Liu J Shen WF Advanced glycation end products induce chemokine/cytokine production via activation of p38 pathway and inhibit proliferation and migration of bone marrow mesenchymal stem cells Cardiovasc Diabetol 2010 9 66 10.1186/1475-2840-9-66 20969783 PMC2987998

[B29] Sun L Ishida T Yasuda T Kojima Y Honjo T Yamamoto Y Yamamoto H Ishibashi S Hirata K Hayashi Y RAGE mediates oxidized LDL-induced pro-inflammatory effects and atherosclerosis in non-diabetic LDL receptor-deficient mice Cardiovasc Res 2009 82 2 371 381 19176597 10.1093/cvr/cvp036

[B30] Kamioka M Ishibashi T Sugimoto K Uekita H Nagai R Sakamoto N Ando K Ohkawara H Teramoto T Maruyama Y Blockade of renin-angiotensin system attenuates advanced glycation end products-mediated signaling pathways J Atheroscler Thromb 2010 17 6 590 600 10.5551/jat.3624 20379053

[B31] Yamagishi S Matsui T Nakamura K Inoue H Takeuchi M Ueda S Fukami K Okuda S Imaizumi T Olmesartan blocks advanced glycation end products (AGEs)-induced angiogenesis in vitro by suppressing receptor for AGEs (RAGE) expression Microvasc Res 2008 75 1 130 134 10.1016/j.mvr.2007.05.001 17560613

[B32] Koka V Wang W Huang XR Kim-Mitsuyama S Truong LD Lan HY Advanced glycation end products activate a chymase-dependent angiotensin II-generating pathway in diabetic complications Circulation 2006 113 10 1353 1360 10.1161/CIRCULATIONAHA.105.575589 16520412 PMC1401500

[B33] Kotani K Caccavello R Sakane N Yamada T Taniguchi N Gugliucci A Influence of Physical Activity Intervention on Circulating Soluble Receptor for Advanced Glycation end Products in Elderly Subjects J Clin Med Res 2011 3 5 252 257 22383913 10.4021/jocmr704w PMC3279487

[B34] Hartog JW Willemsen S van Veldhuisen DJ Posma JL van Wijk LM Hummel YM Hillege HL Voors AA Effects of alagebrium, an advanced glycation endproduct breaker, on exercise tolerance and cardiac function in patients with chronic heart failure Eur J Heart Fail 2011 13 8 899 908 10.1093/eurjhf/hfr067 21669961

[B35] Norton GR Candy G Woodiwiss AJ Aminoguanidine prevents the decreased myocardial compliance produced by streptozotocin-induced diabetes mellitus in rats Circulation 1996 93 10 1905 1912 10.1161/01.CIR.93.10.1905 8635270

[B36] Liu J Masurekar MR Vatner DE Jyothirmayi GN Regan TJ Vatner SF Meggs LG Malhotra A Glycation end-product cross-link breaker reduces collagen and improves cardiac function in aging diabetic heart Am J Physiol Heart Circ Physiol 2003 285 6 H2587 2591 12946933 10.1152/ajpheart.00516.2003

[B37] Little WC Zile MR Kitzman DW Hundley WG O'Brien TX Degroof RC The effect of alagebrium chloride (ALT-711), a novel glucose cross-link breaker, in the treatment of elderly patients with diastolic heart failure J Card Fail 2005 11 3 191 195 10.1016/j.cardfail.2004.09.010 15812746

[B38] Thohan V Koemer MM Pratt CM Torre GA Improvements in diastolic function amond patients with advenced systolic heart failure utilizing alagebrium (an oral advanced glycation end-product cross-link breaker) Circulation 2005 112 Suppl 2 U620 2647

